# Editorial: hypotheses about protein folding - the proteomic code and wonderfolds

**DOI:** 10.1186/1742-4682-6-31

**Published:** 2009-12-24

**Authors:** Paul S Agutter

**Affiliations:** 1 Theoretical Medicine and Biology Group, 26 Castle Hill, Glossop, Derbyshire, SK13 7RR, UK

## Abstract

Theoretical biology journals can contribute in many ways to the progress of knowledge. They are particularly well-placed to encourage dialogue and debate about hypotheses addressing problematical areas of research. An online journal provides an especially useful forum for such debate because of the option of posting comments within days of the publication of a contentious article.

## Editorial

'Theoretical biology' encompasses proposals ranging from new mathematical models of well-studied biological processes to speculative notions that inhabit the borderland between science and philosophy. *Theoretical Biology and Medical Modelling *can accommodate everything within that range subject to peer review and editorial approval. Novel hypotheses addressing phenomena that have defied satisfactory explanation are especially welcome, provided they meet the basic criterion of testability (at least in principle), because they can stimulate debate or excite controversy and are *ipso facto *healthy for science.

Theoretical biology has not enjoyed the status of, say, theoretical physics because biology is primarily a science of particular phenomena rather than general laws. 'Grand theories' in biology seldom prove useful or even tenable, as some widely-discussed instances have shown during the past decade. Nevertheless, there are problematic areas of the life sciences that invite theoretical exploration. Protein folding is an example. Most research in this field, as in others, is empirical and pertains *a fortiori *to only a limited range of polypeptides and/or species (e.g. [1,2]). Broad hypotheses about general mechanisms of protein folding may therefore initiate significant contributions to knowledge.

The 'proteomic code' is one such hypothesis. Its basic claim is that while protein primary structure is encoded in the base sequence of mRNA, the rules for protein folding are encoded in other features of messenger structure. Jan Biró of the Homulus Foundation, Los Angeles, has developed the idea in a recent series of papers [3-6], some of them published in this journal, and has recently published a book that explains it in detail [7]. The proteomic code hypothesis is likely to find support among some workers in the protein structure field, but is equally likely to find powerful opponents.

Biró's point of departure is the well-known redundancy of the genetic code. Studying 81 messengers, he showed [3,4] that mRNA subsequences comprising 1st and/or 3rd codon residues have significantly higher free folding energies than subsequences containing only 2nd residues (p < 0.0001). No such periodically distributed differences in free folding energy were found in intron transcripts. This suggests selection for local secondary structures in RNA coding regions, and these structures resemble the folding profiles of the encoded proteins. In particular, codons synonymous in respect of their encoded amino acids may nevertheless signify differences in protein secondary or tertiary structure. Thus, messengers not only direct the assembly of polypeptides with the correct primary sequence (the genetic code), they also direct the correct folding of those polypeptides (the proteomic code) [5,6].

This concept was first suggested a quarter of a century ago by Biró himself, and independently by Mekler, and was developed in studies by Blalock, Root-Bernstein, Siemion, Miller and others [7]. In 2003, Biró and colleagues published a common periodic table of codons and amino acids, which elaborates the proteomic code hypothesis in specific detail [8]. The idea is strikingly consistent with studies such as those of Chiusano *et al. *[9], who showed that the nucleotide frequencies in second codon positions are remarkably different among coding regions that correspond to different protein secondary structures and to amino acids with different physicochemical properties. It is also broadly compatible with the work of Ikehara and colleagues [10,11] and of Rodin and Rodin [12] on the origin and evolution of the genetic code.

However, some research conflicts with the proteomic code concept. A salient example is the work by Berezovsky and colleagues [13,14], whose emphasis is on polymer physics and on the selection for protein stability that causes preferred polypeptide structures to emerge. These authors have identified structural motifs that they dub 'wonderfolds', which arise repeatedly as native states of stable polypeptides resulting from the mutation and selection of random sequences. They reason that superfamilies with wonderfolds may have played an important part in early evolution. This approach to the study of protein folding has no connection at all with mRNA structure or the distinctive properties of codon bases. It seems likely that Berezovsky and his colleagues would dismiss the proteomic code hypothesis as speculative and unproductive, whereas proponents of the proteomic code may wish to relate 'wonderfolds' to particular recurrent combinations of mRNA codons (which would then, in turn, require explanation).

This is a potentially fruitful arena for continuing debate and discussion. Currently, the main questions seem to be (1) whether either hypothesis satisfactorily explains empirical results such as those in [1,2] and (2) whether the two hypotheses - which at present seem incompatible - can ultimately be reconciled. By fostering the further exploration of these and related questions, theoretical biology journals are in a position to make valuable contributions to knowledge. *Theoretical Biology and Medical Modelling *is particularly well placed in this regard because it provides the option of posting comments on contentious articles within days of their online publication.
